# The Role of HIPEC in Isolated Cytology-Positive Gastric Cancer: Nodal Metastasis Dominates Prognosis, HIPEC Remains Unproven

**DOI:** 10.1245/s10434-025-18743-2

**Published:** 2025-11-25

**Authors:** Kunal Nandy, Amir Parray, Akshay Dhaiya, Amit Chopde, Vishnu Menon, Vikas Ostwal, Anant Ramaswamy, Prabhat Bhargav, Manish S Bhandare, Shailesh V Shrikhande, Vikram A Chaudhari

**Affiliations:** 1 Division of GI and HPB Surgery, Department of Surgical Oncology, Tata Memorial Hospital, Homi Bhabha National Institute, Parel, Mumbai, India; 2 Department of Medical Oncology, Tata Memorial Hospital, Homi Bhabha National Institute, Parel, Mumbai, India; 3 HPB & Upper GI Unit, Department of Surgical Oncology, Tata Memorial Centre - Advanced Centre for Treatment, Research and Education in Cancer (ACTREC), Homi Bhabha National Institute, Mumbai, India

**Keywords:** Gastric cancer, Cytology-positive, HIPEC, Nodal metastasis, Peritoneal recurrence, Propensity-matched, Precision oncology

## Abstract

**Background:**

The therapeutic role of hyperthermic intraperitoneal chemotherapy (HIPEC) in isolated cytology-positive (Cy^+^) gastric cancer without peritoneal metastases (Peritoneal Cancer Index score 0) remains investigational. This study evaluated survival outcomes, prognostic factors, and exploratory HIPEC efficacy in Cy^+^ patients treated with modern multimodal therapy.

**Methods:**

We retrospectively analyzed 558 patients with gastric adenocarcinoma who received neoadjuvant chemotherapy followed by radical gastrectomy (2017–2023). Propensity score matching (1:5) balanced 47 Cy^+^ and 511 cytology-negative (Cy^−^) patients for pathologic stage, treatment response, and adjuvant completion. Inverse probability of treatment weighting validated matched results. Primary endpoints were overall survival (OS) and disease-free survival (DFS).

**Results:**

Cy^+^ patients had inferior outcomes (median OS 24.7 vs. 57.2 months; hazard ratio [HR] 2.09; *p*<0.001; DFS 14.7 vs. 41.4 months; HR 1.62; *p*=0.012), which persisted after matching and inverse probability of treatment weighting. Within the Cy^+^ cohort, nodal metastasis was the strongest prognostic factor, tripling recurrence risk (DFS HR 3.06; *p*=0.018). Among 23 Cy^+^ patients undergoing HIPEC, 2-year DFS was 41.4 versus 29.5% (*p*=0.398) and OS was 55.6 versus 42.4% (*p*=0.903) compared with non-HIPEC cases. HIPEC increased operative time and hospital stay but showed comparable morbidity and no 90-day mortality.

**Conclusion:**

Cytology positivity independently predicted poor survival after curative gastrectomy, but nodal status was the dominant prognostic determinant. HIPEC was well tolerated but conferred no significant survival advantage, underscoring its investigational role pending prospective validation.

**Supplementary Information:**

The online version contains supplementary material available at 10.1245/s10434-025-18743-2.

Gastric cancer persists as a global health challenge. Peritoneal dissemination represents the most common metastatic spread and remains a dominant cause of mortality worldwide.^1–3^ The detection of free intraperitoneal tumor cells without macroscopic disease—designated isolated cytology positivity (Peritoneal Cancer Index score [PCI] 0, Cy+)—defines a biologically distinct subset of stage IV gastric cancer.^4–6^ Although classified as metastatic disease, Cy+ status presents a therapeutic dilemma: prognosis is substantially better than overt carcinomatosis but significantly worse than for their cytology-negative (Cy−) counterparts.^7–9^ This prognostic ambiguity has led to divergent management strategies worldwide.

Western guidelines, including those from the National Comprehensive Cancer Network, classify Cy+ as M1 disease and recommend at least 3 months of systemic chemotherapy, followed by reassessment with recalculation of the PCI and repeat cytology. Surgery ± intraperitoneal chemotherapy is considered only within clinical trials and after multidisciplinary discussion.^10^ In contrast, Eastern protocols increasingly incorporate bidirectional therapy, combining intraperitoneal and intravenous chemotherapy, with conversion gastrectomy offered to responders. Although the PHOENIX-GC trial did not demonstrate superiority of intraperitoneal + intravenous therapy over systemic therapy alone, the multicenter Dragon-01 trial showed that intraperitoneal paclitaxel plus S-1 significantly improved overall survival (OS) compared with intravenous paclitaxel plus S-1, with acceptable toxicity.^11,12^ These evolving strategies have enabled select centers to report 5-year survival exceeding 40% in Cy+ patients undergoing multimodality therapy.^13–15^

Hyperthermic intraperitoneal chemotherapy (HIPEC) has been investigated as an adjunct aimed at eradicating microscopic peritoneal disease. However, evidence regarding its role in gastric cancer with peritoneal metastasis remains conflicting.^13–17^ Although some studies suggest benefit in limited peritoneal carcinomatosis,^16–18^ the role of HIPEC in isolated Cy+ disease is controversial and inadequately defined. Current consensus statements recommend HIPEC only in highly selected patients with PCI <10 after at least 3 months of systemic chemotherapy, and preferably within the context of a clinical trial.^10^ Limitations of the existing evidence include small sample sizes, heterogeneous patient populations, variable HIPEC protocols, and lack of robust randomized controls.^19–21^ Moreover, most studies have not evaluated HIPEC within contemporary multimodal treatment frameworks incorporating effective docetaxel-based triplet regimens.

Against this backdrop, the present study of 558 patients treated at a high-volume tertiary center aimed to (i) validate the prognostic significance of isolated peritoneal cytology positivity in the era of modern systemic therapy, (ii) assess the potential survival impact of HIPEC in this cohort, and (iii) identify outcome predictors to enable precise patient selection.

## Material and Methods

**Fig. 1:**
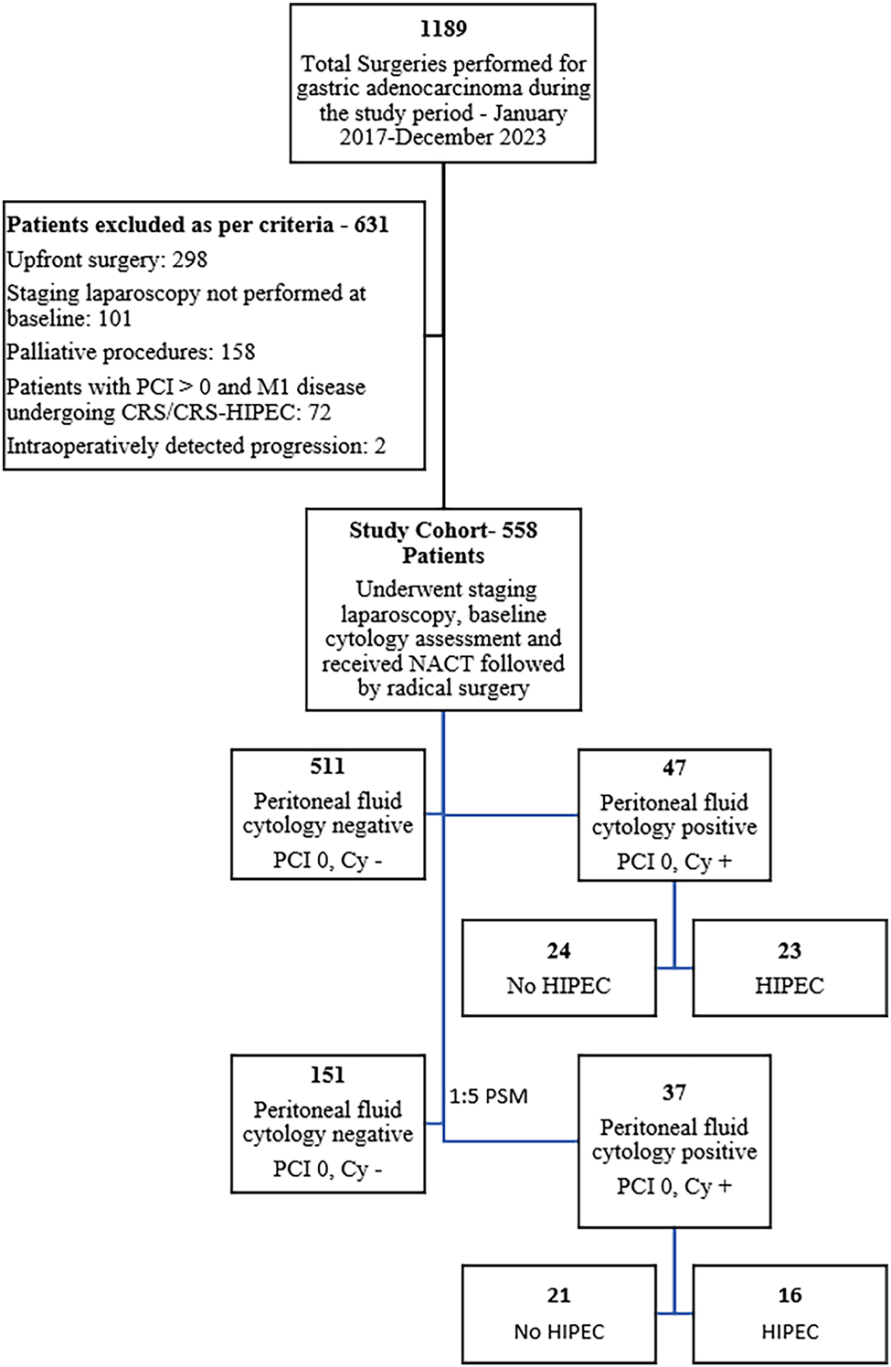
Flow chart illustrating the formation of the study cohort and treatment strategies. CRS, cytoreductive surgery; HIPEC, hyperthermic intraperitoneal chemotherapy; NACT, neoadjuvant chemotherapy; PCI, Peritoneal Cancer Index

This retrospective cohort study analyzed data from a prospectively maintained gastric cancer database at Tata Memorial Hospital (Mumbai, India). Patients undergoing curative-intent radical gastrectomy after neoadjuvant chemotherapy (NACT) between January 2017 and December 2023 were included, following institutional review board approval (IRB-4811) and adherence to the Declaration of Helsinki. Eligible patients had histologically confirmed gastric adenocarcinoma with baseline staging laparoscopy confirming either isolated peritoneal cytology positivity (Cy1; PCI 0) or negative cytology (Cy0). Exclusion criteria included macroscopic peritoneal metastasis (PCI > 0), palliative resection, upfront surgery, or non-peritoneal metastases (Fig. 1).

All patients underwent standardized staging, including triple-phase contrast-enhanced computed tomography (thorax/abdomen/pelvis) and diagnostic laparoscopy. Peritoneal washing cytology was performed by instilling 150–200 mL saline into subdiaphragmatic and pelvic spaces, with cytological positivity defined by unequivocal adenocarcinoma cells on Papanicolaou-stained specimens. In line with institutional practice and to avoid ambiguity, atypical or suspicious cells were also classified as Cy+.

NACT consisted of platinum/fluoropyrimidine-based regimens (DOF: docetaxel 50 mg/m^2^, oxaliplatin 85 mg/m^2^, 5-FU 1200 mg/m^2^; FLOT: 5-FU 2600 mg/m^2^, leucovorin 200 mg/m^2^, oxaliplatin 85 mg/m^2^, docetaxel 50 mg/m^2^) administered every 2 weeks. Treatment duration (median 4 cycles, range 1–12) was determined by multidisciplinary team (MDT) assessment of radiological and clinical response.

Surgical interventions included D2 lymphadenectomy, with the extent of gastrectomy (total, subtotal, or proximal) dictated by tumor location. HIPEC was administered per MDT consensus using cisplatin (75 mg/m^2^) and mitomycin C (25 mg/m^2^) in 3 L peritoneal perfusate at 42–43°C for 60 minutes immediately post-resection. The decision to perform HIPEC was shaped by the gradual implementation of HIPEC services at our center, beginning in 2017. During 2017–2019, HIPEC was not routinely available, and several patients did not receive HIPEC because of this limited access. As institutional availability improved, HIPEC was offered more frequently, creating a natural intervention arm. Selection was guided by MDT consensus and influenced by patient age, performance status, surgical complexity, intraoperative blood loss, institutional logistics, machine availability, and surgeon discretion. This relatively balanced distribution allowed exploratory comparison of HIPEC outcomes.

Primary outcomes were OS (time from diagnosis to death or last follow-up) and disease-free survival (DFS; time from surgery to recurrence or last follow-up). Secondary outcomes included 90-day mortality, major morbidity (Clavien–Dindo grade ≥IIIa), and hospital stay. Follow-up comprised clinical and radiological assessments quarterly for 2 years, biannually until 5 years, and annually thereafter. Recurrence required histological or radiological confirmation.

To address potential selection bias between Cy+ and Cy- cohorts, propensity score matching was performed in a 1:5 ratio. Propensity scores were generated through logistic regression incorporating clinically relevant covariates: number of neoadjuvant chemotherapy cycles, pathologic T stage (ypT), pathologic N stage (ypN), perineural invasion status, pathological complete response, and completion of planned adjuvant treatment. Greedy nearest-neighbor matching without replacement was implemented with a caliper width of 0.1 standard deviations of the logit of the propensity score using the *MatchIt* package (version 4.5.0) in R software. In addition, inverse probability of treatment weighting (IPTW) was performed as a sensitivity analysis to validate the robustness of the propensity-matched findings.

Categorical variables were compared using *χ*^2^ or Fisher's exact tests, whereas continuous variables were assessed with Mann–Whitney U or *t*-tests as appropriate. Survival analysis employed Kaplan–Meier methodology with log-rank testing. Cox proportional hazards models identified prognostic factors, with variables with a *p*<0.10 in univariate analysis entered into multivariate modelling. All analyses were performed using SPSS Statistics v25.0 (IBM Corp.) and R v4.3.2 (R Foundation), with statistical significance set at *p*<0.05 (two-tailed).

## Results

### Cohort Characteristics and Treatment Patterns

A total of 1189 gastrectomies were performed between January 2017 and December 2023. After applying inclusion criteria, 558 patients formed the analytical cohort (Fig. 1). The median age was 55 years (range 18–85) with male predominance (69%). Baseline staging laparoscopy identified isolated peritoneal Cy+ in 47 patients (8.4%), and 511 patients (91.6%) had Cy-. Clinicopathological characteristics were well balanced between groups, except Cy+ patients more frequently underwent total gastrectomy (48.9 vs. 34.0%, *p*=0.034) and received HIPEC (48.9% of Cy+ cohort) [Table 1]. Neoadjuvant chemotherapy predominantly featured DOF-based regimens (75.4%), with a median of 4 cycles administered. The median interval from diagnosis to surgery was 18 weeks (interquartile range 15–22), with no significant difference between cytology groups (*p*=0.214).

**Table 1:** The clinical characteristics and pathological outcomes

Parameters		Overall population (*n*=558)		Peritoneal fluid Cy− (*n*=511) (PCI 0, Cy−)		Peritoneal fluid Cy+ (*n*=47) (PCI 0, Cy+)	*P*-value
Median age		55 (18–85)		55 (18–83)		55 (27–85)	0.960
Sex	Male	385 (69)		353 (69)		32 (68)	
	Female	173 (31)		158 (31)		15 (32)	0.88
BMI, kg/m^2^		21.5 (13–39.1)		21.6 (13–39.1)		19.8 (13–30.9)	0.067
Disease location	Gastroesophageal junction/body/proximal	281 (50.3)		255 (49.9)		26 (55.3)	
	Distal stomach/antrum	252 (45.2)		232 (45.4)		20 (42.5)	0.653
	Linitis plastica	25 (4.5)		24 (4.6)		1 (2.1)	
NACT	DOF based	421 (75.4)		381 (74.5)		40 (85.1)	
	Non-DOF based	137 (24.6)		130 (25.5)		7 (14.9)	0.108
Median number of cycles		4 (1–12)		4 (1–12)		4 (3–9)	**0.001**
>4 cycles NACT		431 (77.2)		391 (76.5)		40 (85.1)	0.179
8 cycles NACT		23 (4.1)		8 (23.5)		15 (31.9)	**0.001**
Surgery	Total gastrectomy	197 (35.3)		174 (34)		23 (48.9)	
	Subtotal gastrectomy	311 (55.7)		289 (56.5)		22 (46.8)	
	Proximal gastrectomy	29 (5.2)		28 (5.4)		1 (2.1)	0.276
	Esophagogastrectomy	8 (1.4)		8 (1.5)		0	
	Multivisceral resection	13 (2.3)		12 (2.3)		1 (2.1)	
Median surgery duration, minutes		360 (160–660)		340 (160–600)		360 (160–660)	0.871
Median blood loss, mL		800 (200–2800)		800 (250–2200)		800 (200–2800)	0.761
Major morbidity (>3CD A)		53 (9.4)		47 (9.1)		6 (12.7)	0.091
90-day mortality		4 (0.7)		4 (0.7)		0	
Median hospital stay, days		8 (4–55)		8 (4–55)		9 (4–39)	0.150
Tumor differentiation	Well/moderate	188 (33.7)		170 (33.2)		18 (38.2)	0.485
	Poor	370 (66.3)		341 (66.7)		29 (61.7)	
ypT-stage	T0/T1/T2	244 (43.7)		210 (41.1)		34 (72.3)	
	T3/T4	314 (56.3)		301 (58.9)		13 (27.6)	**0.001**
ypN-stage	N0	251 (45)		234 (45.7)		17 (36.1)	
	N+	307 (55)		277 (54.2)		30 (63.8)	0.204
Lymph node yield		27 (3–89)		27 (3–89		31 (12–66)	0.098
Margin	R0	535 (95.9)		490 (95.8)		45 (95.7)	
	R+	23 (4.1)		21 (4.2)		2 (4.3)	0.962
LVI	Present	180 (32.3)		164 (32.1)		16 (34.1)	
	Absent	378 (67.7)		347 (67.9)		31 (65.9)	0.784
PNI	Present	143 (25.6)		124 (24.2)		19 (40.4)	
	Absent	415 (74.4)		387 (75.7)		28 (59.5)	**0.015**
PCR		50 (9)		42 (8.2)		8 (17)	**0.043**
TRG	1,2	138 (24.7)		123 (24.1)		15 (31.9)	
	3,4,5	420 (75.3)		388 (75.9)		32 (68.1)	0.233
Completed adjuvant regimen		529 (94.8%)		487 (95.3)		42 (89.3)	0.079
Recurrence	Local	38 (21)		36 (7)		2 (4.2)	
	Distant	133 (73.5)		118 (23)		15 (31.9)	0.550
	Both	10 (5.5)		9 (1.7)		1 (2.1)	
Peritoneal recurrence	Yes	77 (42.5)		67 (13.1)		10 (21.2)	0.239
	No	104 (57.5)		96 (18.7)		8 (17)	

Data are presented as median (interquartile range) or N (%) unless otherwise indicated.

BMI, body mass index; CD, Clavien–Dindo; DOF, docetaxel, oxaliplatin, and 5-flurouracil; LVI, lymphovascular invasion; NACT, neoadjuvant chemotherapy; PCR, pathological complete response; PNI, perineural invasion; TRG, tumor regression grading

### Survival Outcomes Stratified by Cytology Status

**Fig. 2:**
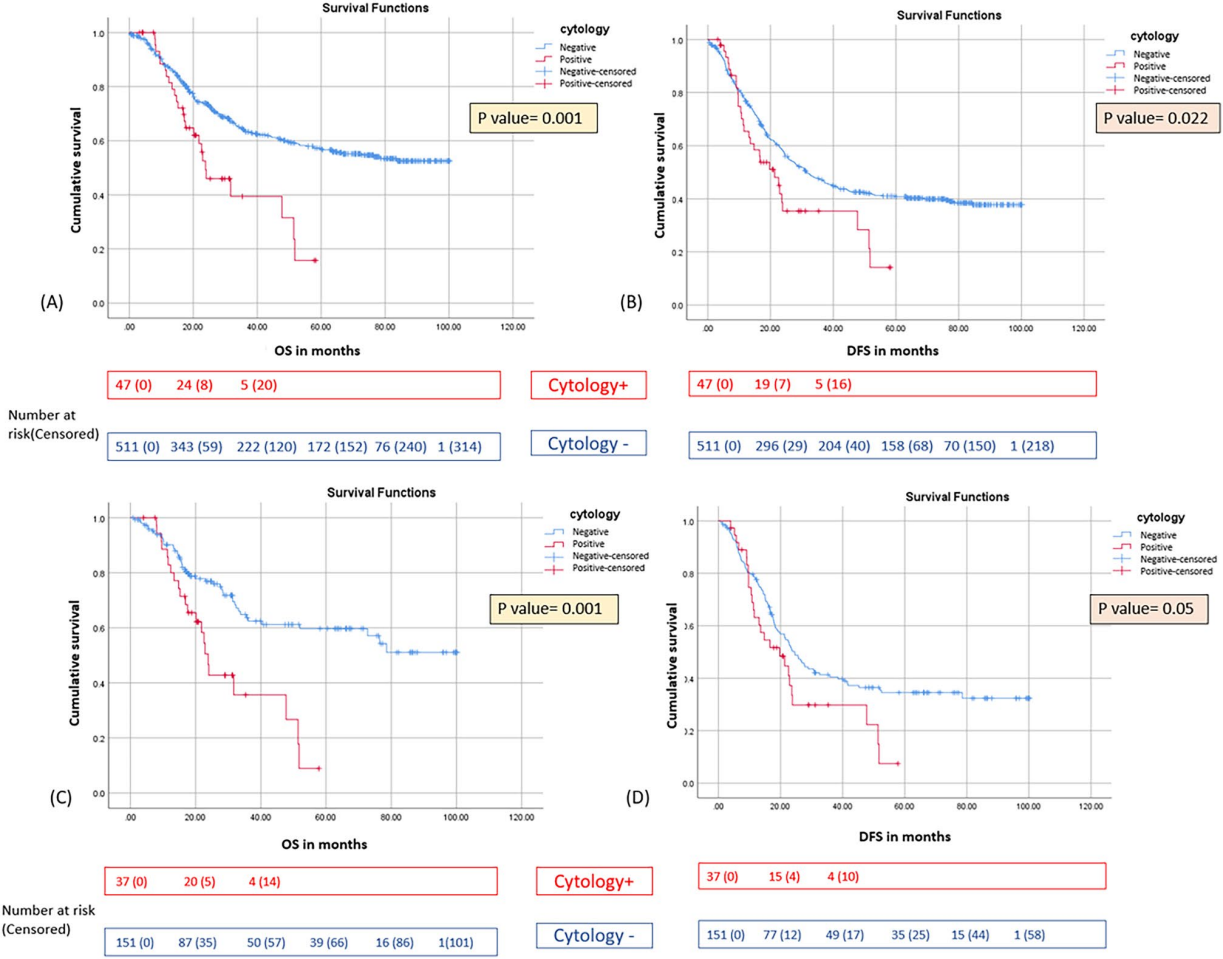
Kaplan–Meier curve demonstrating the effect of peritoneal fluid cytology on **A** overall survival (OS) and **B** disease-free survival (DFS). Kaplan–Meier curve demonstrating the effect of peritoneal fluid cytology on **C** OS and **D** DFS in the matched population

At median follow-up of 57.3 months (95% confidence interval [CI] 52.1–62.5), cytology status was confirmed as an adverse prognostic factor. Cy+ patients had significantly inferior median OS (24.7 vs 57.2 months; HR 2.09; 95% CI 1.37–3.18; *p*<0.001) and DFS (14.7 vs 41.4 months; HR 1.62; 95% CI 1.11–2.36; *p*=0.012) than did their Cy- counterparts (Table 2). These differences persisted after propensity score matching (1:5 ratio) (Table 3). In the matched cohort (37 Cy+ vs. 151 Cy−), Cy+ patients retained significantly worse survival: median OS 23.8 months versus not reached (*p*=0.001) and median DFS 19.7 versus 24.5 months (*p*=0.05). The 3-year OS was 38.3% in Cy+ versus 68.7% in Cy− patients (p<0.001), confirming cytology status as an independent negative prognostic marker (Fig. 2A–D). Sensitivity analysis using IPTW demonstrated results consistent with the propensity-matched cohort, reinforcing the robustness of these findings (see the supplementary file). The HR for OS was 2.81 (95% CI 1.86–4.25), *p*<0.001 and for DFS was 1.98 (95% CI 1.31–2.98), *p*<0.001.

**Table 2:** Univariate and multivariate analysis for overall survival (OS) and disease-free survival (DFS)

		OS				DFS			
		Univariate analysis		Multivariate analysis		Univariate analysis		Multivariate analysis	
Parameter		HR (95% CI)	*P* value	HR (95% CI)	*P* value	HR (95% CI)	*P* value	HR (95% CI)	*P* value
Age		1.01 (0.99–1.022)	0.078			1.003 (0.994–1.013)	0.496		
Sex	Male	1.07 (0.80–1.41)	0.638			0.974 (0.770–1.23)	0.828		
	Female	**1**				1			
Peritoneal fluid cytology	Positive	**2.09 (1.37–3.18)**	**0.001**	**2.002 (1.30–3.07)**	**0.002**	**1.62 (1.11–2.36)**	**0.012**	**1.47 (1.007–2.16)**	**0.046**
	Negative	1		1		1		1	
NACT	DOF based	1		**1**		1			
	Non-DOF based	**1.56 (1.18–2.06)**	**0.002**	**1.44 (1.009–1.91**					
**0**	**0.010**	1.15 (0.90–1.48)	0.24						
>4 cycles NACT	Yes	**0.70 (0.53–0.94)**	**0.017**	1.01 (0.68–1.51)	0.935	0.99 (0.76–1.28)	0.953		
	No	1		1		1			
Tumor differentiation	Well/Moderate	**1**		1		1			
	Poor	**1.34 (1.01–1.80)**	**0.047**	1.12 (0.816–1.55)	0.470	1.02 (0.812–1.28)	0.847		
pT-stage	T0/T1/T2	**1**		1		**1**		**1**	
	T3/T4	**4.02 (2.79–5.80)**	**0.001**	**2.70 (1.82–3.98)**	**0.001**	**2.714 (2.08–3.52)**	**0.001**	**1.99 (1.50–2.64)**	**0.001**
pN-stage	N0	**1**		**1**		**1**		**1**	
	N+	**3.18 (2.36–4.30)**	**0.001**	**2.34 (0.77–2.3)**	**0.001**	**2.61 (2.05–3.29)**	**0.001**	**2.06 (1.61–2.63)**	**0.001**
Margin	R0	**1**		1		**1**		1	
	R+	**2.50 (1.50–4.160**	**0.001**	1.377 (0.798–2.377)	0.251	**1.89 (1.17–3.04)**	**0.009**	1.32 (1.07–1.75)	0.254
LVI	Present	**2.25 (1.72–2.93)**	**0.001**	1.21 (0.89–1.61)	0.290	**1.90 (1.52–2.38)**	**0.001**	1.13 (0.88–1.45)	0.317
	Absent	**1**		21		**1**		1	
PNI	Present	**2.23 (1.69–2.96)**	**0.001**	1.21 (0.98–1.76)		**1.97 (1.56–2.48)**	**0.001**	**1.36 (1.07–1.75)**	**0.013**
	Absent	**1**		1			**1**		
PCR	Yes	**0.22 (0.1–0.50)**	**0.001**	1.15 (0.46–2.85)	0.756	**0.321 (0.184–0.559)**	**0.001**	1.21 (0.65–2.24)	0.542
	No	**1**		1		1		1	
Completed adjuvant regimen	Yes	**0.46 (0.27–0.76)**	**0.003**	**0.53 (0.31–0.89)**	**0.016**	0.639 (0.397–1.08)	0.065		
	No	**1**		**1**		1			
Signet	Yes	**1.51 (1.14–2.01)**	**0.004**	1.32 (0.99–1.77)	0.053	1.22 (0.95–1.56)	0.107		
	No	**1**							

CI, confidence interval; DOF, docetaxel, oxaliplatin, and 5-flurouracil; HR, hazard ratio; LVI, lymphovascular invasion; NACT, neoadjuvant chemotherapy; PCR, pathological complete response; PNI, perineural invasion; TRG, tumor regression grading.

**Table 3:** Characteristics between cytology-positive (Cy+) and cytology-negative (Cy−) patients after propensity matching

Median number of NACT cycles		Cy+ (*n*=37)	Cy− (*n*=151)	*P* value
		4 (3-8)	4 (3-8)	0.136
Tumor differentiation	Well/Moderate	16 (43.2)	56 (37.1)	0.490
	Poor	21 (56.7)	95 (62.9)	
ypT-stage	T0/T1/T2	24 (64.8)	93 (61.5)	0.713
	T3/T4	13 (35.1)	58 (38.4)	
ypN-stage	N0	13 (35.1)	54 (35.7)	0.943
	N+	24 (64.8)	97 (64.2)	
PNI	Present	13 (35.1)	54 (35.7)	0.943
	Absent	24 (64.8)	97 (64.2)	
PCR		4 (10.8)	16 (10.5)	0.970
Completed adjuvant regimen		34 (91.8)	143 (94.7)	0.514

NACT, neoadjuvant chemotherapy; PCR, pathological complete response; PNI, perineural invasion

### HIPEC Outcomes in Cy+ Disease

**Fig. 3:**
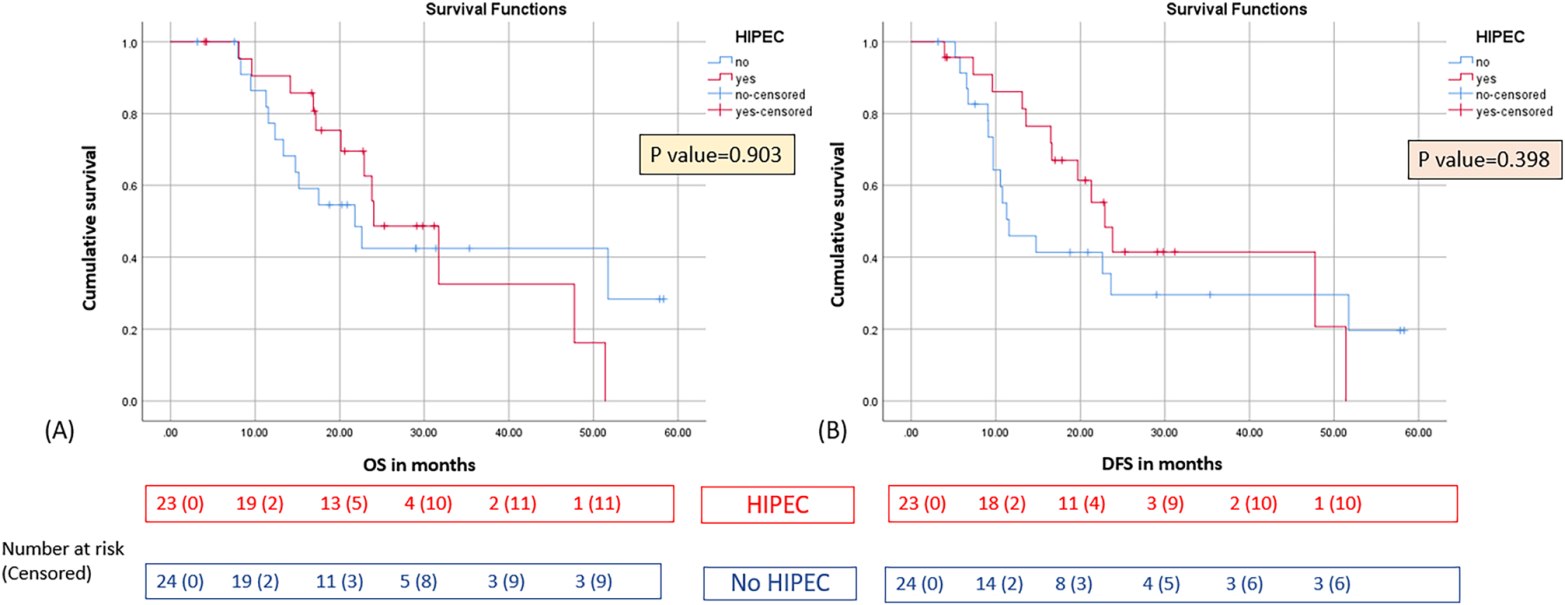
Kaplan–Meier curve demonstrating the effect of hyperthermic intraperitoneal chemotherapy (HIPEC) on **A** overall survival and **B** disease-free survival. All procedures were in accordance with the ethical standards of the responsible committee on human experimentation (institutional and national) and with the Helsinki Declaration of 1964 and later versions. Informed consent to be included in the study, or the equivalent, was obtained from all patients

Among 47 Cy+ patients, 23 (48.9%) underwent HIPEC during radical gastrectomy. HIPEC did not confer a statistically significant survival advantage. The HIPEC group demonstrated an 11.9% higher 2-year DFS (41.4 vs. 29.5%; HR 0.72; 95% CI 0.34–1.53; *p*=0.398) and a 13.2% higher 2-year OS (55.6 vs. 42.4%; HR 0.95; 95% CI 0.42–2.13; *p*=0.903), but neither difference reached significance (Fig. 3A–B). Thus, observed trends should be regarded as hypothesis-generating only. These outcomes occurred despite significantly longer operative times (median 480 vs. 300 minutes; p<0.001) and hospital stays (median 11 vs 9 days; p=0.018) in the HIPEC group. Major morbidity rates (Clavien–Dindo ≥IIIa) were comparable (13.0% vs. 12.5%; p=0.955), as was blood loss (median 850 mL vs. 575 mL; p=0.091). Importantly, there was no 90-day mortality in either group, underscoring the procedural safety of HIPEC.

### Determinants of Prognosis in High-Risk Subgroups

Multivariable analysis of the Cy+ cohort confirmed nodal status as the dominant prognostic factor (Table 4). Node-positive disease significantly worsened survival (OS HR 2.86; 95% CI 1.13–7.25; *p*=0.026; DFS HR 3.06; 95% CI 1.21–7.73; *p*=0.018). Margin status also independently affected outcomes, with R1 resection increasing mortality risk 15-fold (HR 14.96; 95% CI 1.34–166.18; *p*=0.028). Pathological complete response showed protective trends (DFS HR 0.39, *p*=0.430) but without statistical significance. Perineural invasion, though significant on univariate analysis, did not remain significant in multivariate analysis.

**Table 4:** Univariate and multivariate analysis for overall survival (OS) and disease-free survival (DFS) in Peritoneal Cancer Index score (PCI) 0, cytology positive (Cy+) patients (*N*=47)

Parameter		Overall survival					Disease-free survival					
		Univariate ajlvtanalysis		Multivariate analysis			Univariate analysis			Multivariate analysis		
		HR (95% CI)	*P* value	HR (95% CI)		*P* value	HR (95% CI)		*P* value	HR (95% CI)		*P* value
Age		0.99 (0.96–1.02)	0.56				**0.99 (0.96–1.02)**		**0.69**			
Sex	Male	0.69 (0.31–1.55)	0.374				0.69 (0.32–1.47)		0.343			
	Female	1					1					
NACT	DOF based	1					1					
	Non-DOF based	2.15 (0.78–5.92)	0.138				2.25 (0.82–6.18)		0.113			
>4 cycles NACT	Yes	1.07 (0.317–3.68)	0.903				1.18 (0.35–3.96)		0.78			
	No	1					1					
8 cycles NACT	Yes	1.01 (0.435–2.35)	0.978				0.818 (0.36–1.85)		0.63			
	No	1					1					
Tumor differentiation	Well/moderate	1					1					
	Poor	1.20 (0.51–2.83)	0.665				1.18 (0.54–2.59)		0.66			
pT-stage	T0/T1/T2	**1**		1			**1**			1		
	T3/T4	**2.6 (0.97–7.009)**	**0.05**	2.02 (0.73–5.58)		0.175	**2.915 (1.18–7.20)**		**0.02**	2.18 (0.86–5.54)		0.099
pN-stage	N0	**1**		**1**			**1**			**1**		
	N+	**2.98 (1.18–7.50)**	**0.020**	**2.86 (1.13–7.25)**		**0.026**	**3.73 (1.51–9.21)**		**0.004**	**3.06 (1.21–7.73)**		**0.018**
Margin	R0	1		1			1			1		
	R+	**20.49 (1.85–226.04)**	**0.014**	**14.96 (1.34–166.18)**		**0.028**	**10.3 (1.15–92.46)**		**0.037**	6.29 (0.69–56.81)		0.101
LVI	Present	2.04 (0.93–4.52)	0.075				**2.15 (1.02–4.54)**		**0.043**	**1.04 (0.41–2.64)**		**0.922**
	Absent	1					1					
PNI	Present	1.42 (0.63–3.16)	0.390				1.93 (0.92–4.04)		0.082			
	Absent	1					1			1		
PCR	Yes	0.17 (0.024–1.31)	0.091				**0.125 (0.017–0.922)**		**0.041**	0.394 (0.039–3.97)		0.430
	No	1					**1**					
Completed adjuvant regimen	Yes	0.58 (0.16–1.98)	0.38				0.913 (0.273–3.05)		0.88			
	No	1					1					
HIPEC	Yes	0.95 (0.42–2.13)	0.903				1					
	No	1					0.724 (0.34–1.53)		0.40			

CI, confidence interval; DOF, docetaxel, oxaliplatin and 5-flurouracil; HIPEC, hyperthermic intraperitoneal chemotherapy; HR, hazard ratio; LVI, lymphovascular invasion; NACT, neoadjuvant chemotherapy; PCR, pathological complete response; PNI, perineural invasion; TRG, tumor regression grading.

### Patterns of Treatment Failure

Recurrence analysis revealed higher rates of peritoneal recurrence in Cy+ than in Cy− patients (21.2 vs. 13.1%; *p*=0.239), although this was not statistically significant (Table 1). Median time to recurrence was shorter in Cy+ patients (8.3 vs. 14.6 months; *p=*0.008). In Cy+ patients treated with HIPEC, peritoneal recurrence rates were numerically lower (34.8 vs. 50.0%; *p*=0.287), but again without statistical significance. Locoregional recurrences were infrequent (<6%) across all groups, confirming adequacy of surgical clearance.

## Discussion

This large contemporary analysis of isolated peritoneal Cy+ gastric cancer (PCI 0) confirms that Cy+ status independently doubles mortality risk (HR 2.09; p<0.001) despite modern multimodal therapy. Cy+ patients experienced a 50% shorter median survival than stage-matched Cy– counterparts (3-year OS 38.3% vs. 68.7%; *p*<0.001), underscoring an intrinsic biological aggression beyond conventional staging. Consistent with Kodera’s report of a 23-month median OS in Cy+ patients,^20^ our study reinforces the adverse biological impact of free peritoneal tumor cells, likely mediated by processes such as epithelial–mesenchymal transition and immune evasion,^5,22^ which remain insufficiently targeted by current therapies.

A key insight is that nodal metastasis emerged as the strongest prognostic factor within the Cy+ cohort. Any nodal involvement raised the recurrence hazard three-fold (DFS HR 3.06; *p*=0.018), exceeding the risk attributable to cytology alone. Rather than overturning peritoneal-centric models, this refines risk stratification by showing that nodal disease accelerates systemic dissemination and compounds peritoneal relapse. Although Shim et al.^23^ highlighted N3 status as prognostic, our data demonstrate that even N1–N2 involvement significantly worsened outcomes. Recent multi-institutional work further underscores nodal burden as a marker of tumor biology in gastric peritoneal disease.^24^ Margin status also independently influenced survival, reinforcing the necessity of R0 resection in this high-risk group.^25^

The study also offers the largest direct comparison of HIPEC versus no HIPEC in isolated Cy+ gastric cancer. Earlier series reporting favorable HIPEC outcomes in this subgroup were limited by small cohorts and lacked appropriate control groups.^15–17,27,30^ In our matched Cy+ cohort (*n*=23 HIPEC vs. *n*=24 non-HIPEC), 2-year DFS was 41.4% with HIPEC versus 29.5% without, and 2-year OS was 55.6 versus 42.4%; neither difference reached statistical significance (DFS *p*=0.398; OS *p*=0.903). These findings confirm that, although HIPEC is technically feasible and safe, its survival benefit remains unproven. Consistent with Li et al.^26^, where conversion of cytology to negative following chemotherapy improved prognosis, our results reinforce the primacy of systemic therapy. Accordingly, observed HIPEC trends should be interpreted as hypothesis-generating, not practice-changing.

Methodological limitations merit acknowledgement. Limitations include the retrospective design with inherent selection bias, and HIPEC allocation influenced by availability and intraoperative factors. Exclusion of non-operative patients may have inflated survival outcomes, and lack of post-NACT cytology reassessment limited our evaluation of cytology conversion.^25,27–29^ The small Cy+ sample (*n*=47) further reduced power to detect moderate survival differences. These factors underscore the need for prospective, biomarker-driven trials using optimized intraperitoneal strategies. Despite these limitations, the study has notable strengths. It represents one of the largest single-institution cohorts of isolated Cy+ gastric cancer treated with contemporary multimodal therapy and D2 gastrectomy. The phased institutional implementation of HIPEC enabled the formation of an intervention cohort and facilitated comparison in a real-world clinical setting. Rigorous propensity score matching, IPTW sensitivity analysis, long median follow-up and detailed recurrence profiling enhances the statistical robustness and clinical relevance of survival comparisons.

Overall, isolated Cy+ remains a poor prognostic indicator. Within this subgroup, nodal metastasis further refines risk stratification, nearly tripling recurrence and mortality risk. Given that HIPEC did not confer a statistically significant survival benefit, emphasis should remain on systemic therapy intensification—whether through enhanced perioperative regimens, maintenance strategies, or integration of immunotherapy. The ongoing GASTRICHIP study is expected to provide more definitive evidence. Until then, radical surgery achieving R0 resection and tailored systemic therapy remain the cornerstone of management, with HIPEC reserved for clinical trial settings.

## Conclusion

Cy+ remains an independent adverse prognostic factor in gastric cancer, but nodal status is the dominant survival determinant in Cy+ patients. HIPEC showed no statistically significant benefit despite numerical trends and should remain investigational. Optimal outcomes depend on R0 resection and systemic therapy tailored to nodal burden. Future trials should explore HIPEC within biomarker-guided, immunotherapy-integrated frameworks.

## Supplementary Information

Below is the link to the electronic supplementary material.

Supplementary file1 (DOCX 302 KB)
