# Supplementary material for: The Role of HIPEC in Isolated Cytology-Positive Gastric Cancer: Nodal Metastasis Dominates Prognosis, HIPEC Remains Unproven
Source: Ann Surg Oncol. 2025 Nov 25;33(4):2960–71. doi: 10.1245/s10434-025-18743-2 (PMC12982269; doi:10.1245/s10434-025-18743-2)
Supplement: Supplementary file 1 — Supplementary file1 (DOCX 302 KB) [file 10434_2025_18743_MOESM1_ESM.docx]

Supplementary Figure: Sensitivity analysis with IPTW for Overall survival


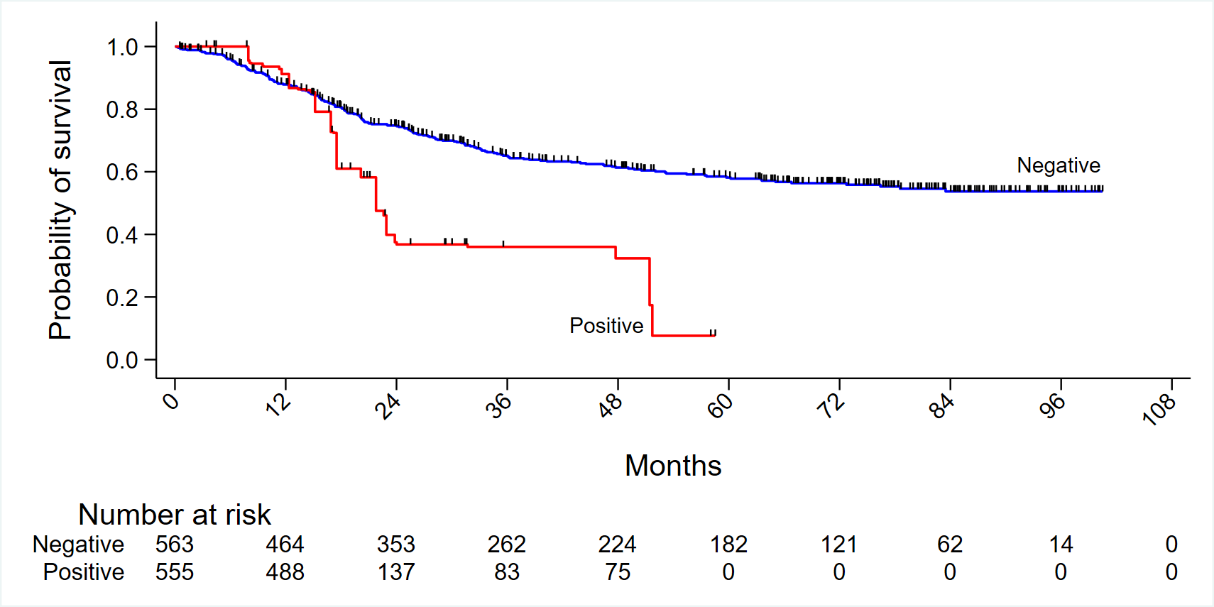


| Group | Time | No. of Events/No. of Patients (%) | Survival Probability | HR [95% CI] | P Value‡ | Log Rank Statistic | P Value† |  |
| --- | --- | --- | --- | --- | --- | --- | --- | --- |
| Negative | 12 | 208/563 (37) | 87.88 | 1 (Ref) | <0.001 | 3.51 | <0.001 |  |
|  | 36 |  | 65.17 |  |  |  |  |  |
|  | 60 |  | 58.15 |  |  |  |  |  |
| Positive | 12 | 364/555 (66) | 91.2 | 2.81 [1.86 to 4.25] |  |  |  |  |
|  | 36 |  | 36.01 |  |  |  |  |  |
|  | 60 |  | - |  |  |  |  |  |
| ‡ P Value calculated by weighted Cox Regression | | | |  |  |  |  |  |
| † P Value calculated by Log Rank test adjusted with IPTW | | | | |  |  |  |  |


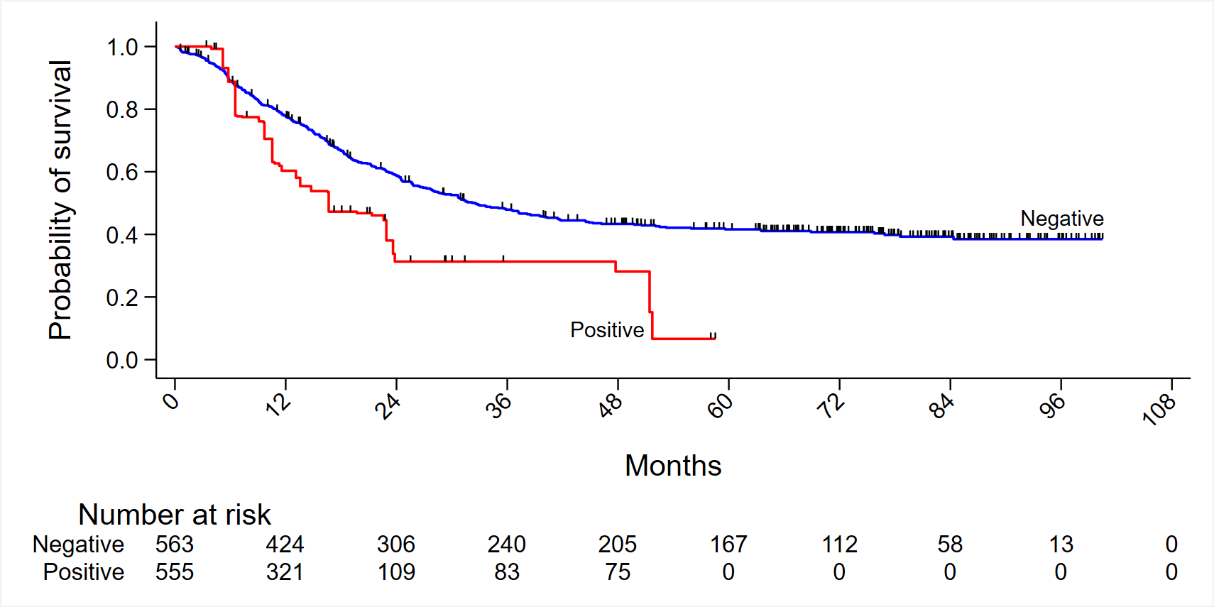
Supplementary Figure: Sensitivity analysis with IPTW for DFS

| Group | Time | No. of Events/No. of Patients (%) | Survival Probability | HR [95% CI] | P Value‡ | Log Rank Statistic | P Value† |
| --- | --- | --- | --- | --- | --- | --- | --- |
| Negative | 12 | 316/563 (56) | 77.79 | 1 (Ref) | 0.001 | 2.29 | 0.022 |
|  | 36 |  | 47.91 |  |  |  |  |
|  | 60 |  | 41.62 |  |  |  |  |
| Positive | 12 | 405/555 (73) | 60.32 | 1.98 [1.31 to 2.98] |  |  |  |
|  | 36 |  | 31.33 |  |  |  |  |
|  | 60 |  | - |  |  |  |  |
| ‡ P Value calculated by weighted Cox Regression | | | |  |  |  |  |
| † P Value calculated by Log Rank test adjusted with IPTW | | | | |  |  |  |
